# Categorizing and Harmonizing Natural, Technological, and Socio-Economic Perils Following the Catastrophe Modeling Paradigm

**DOI:** 10.3390/ijerph191912780

**Published:** 2022-10-06

**Authors:** Arnaud Mignan

**Affiliations:** 1 Institute of Risk Analysis, Prediction and Management (Risks-X), Academy for Advanced Interdisciplinary Studies, Southern University of Science and Technology (SUSTech), Shenzhen 518055, China; mignana@sustech.edu.cn; 2 Department of Earth and Space Sciences, Southern University of Science and Technology (SUSTech), Shenzhen 518055, China

**Keywords:** multi-hazard, standardization, power-law, extreme value, analytical expression, cellular automaton, agent-based model, energy metric

## Abstract

The literature on probabilistic hazard and risk assessment shows a rich and wide variety of modeling strategies tailored to specific perils. On one hand, catastrophe (CAT) modeling, a recent professional and scientific discipline, provides a general structure for the quantification of natural (e.g., geological, hydrological, meteorological) and man-made (e.g., terrorist, cyber) catastrophes. On the other hand, peril characteristics and related processes have yet to be categorized and harmonized to enable adequate comparison, limit silo effects, and simplify the implementation of emerging risks. We reviewed the literature for more than 20 perils from the natural, technological, and socio-economic systems to categorize them by following the CAT modeling hazard pipeline: (1) event source → (2) size distribution → (3) intensity footprint. We defined the following categorizations, which are applicable to any type of peril, specifically: (1) point/line/area/track/diffuse source, (2) discrete event/continuous flow, and (3) spatial diffusion (static)/threshold (passive)/sustained propagation (dynamic). We then harmonized the various hazard processes using energy as the common metric, noting that the hazard pipeline’s underlying physical process consists of some energy being transferred from an energy stock (the source), via an event, to the environment (the footprint).

## 1. Introduction

**Figure 1:**
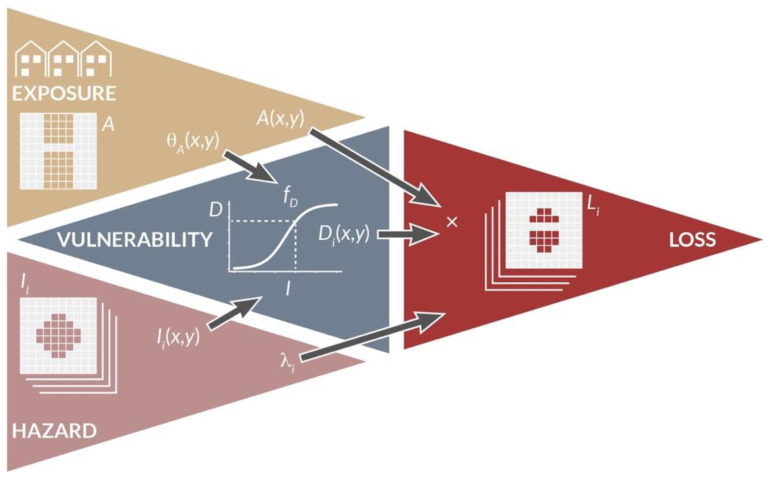
The CAT modeling framework. The binary grids represent the different spatial footprints for the exposed assets and for the stochastic events i. See text for details and notation.

Catastrophe (CAT) modeling has developed in the past 40 years from pilot applications [1] to the standard that it is today, used by insurers and reinsurers, public agencies, and corporations [2,3,4]. The process is a computational pipeline (Figure 1) where a mapping is performed between hazard intensity I(x,y) and loss L(x,y) via a vulnerability function $f_{D}$. It is defined as

(1) $$L=A\cdot f_{D}\left(I,\theta_{A}\right),$$

where A is the exposed asset value, $\theta_{A}$ the asset exposure characteristics, and $D=f_{D}\left(I,\theta_{A}\right)$ the damage level, such as the mean damage ratio (MDR). Equation (1) represents the Risk Triangle with L depending on the following three elements: exposure A, vulnerability $f_{D}$, and hazard I [5]. For a given event i, losses are aggregated for all the geographical locations of coordinates (x,y) impacted by the event, with $L_{i}=\underset{x}{\sum }\underset{y}{\sum }L\left(x,y\right)$ the total event loss. This mapping is repeated for each event for a specific region and peril, typically an earthquake, a storm, or a flood. Considering a set of n stochastic events yields the event loss table (ELT) that lists the event identifier i, its rate $\lambda_{i}$, and its loss $L_{i}$. Risk metrics can then be computed from the ELT using various actuarial methods for portfolio analysis, including the average annual loss (AAL) and exceedance probability (EP) curves [2,4]. At each step of the process, uncertainties can be implemented via various statistical distributions. Site effects and other correcting factors can also be added at any location (x,y). The Monte Carlo method can also be used to simulate a year loss table (YLT) which provides more flexibility in the treatment of uncertainties, the aggregation of losses, and the inclusion of processes beyond the homogeneous Poisson process (e.g., clustering [6], seasonality [7], cascading effects and other loss amplifying factors [8]). For the interested reader, more details can be found in [4].

The probabilistic risk assessment part is standardizable across different perils since a common damage metric can consistently be used (e.g., the MDR). Moreover, vulnerability functions could, in theory, be developed following similar engineering methods independently of the hazard stressor type [9]. It is only the probabilistic hazard assessment component that remains peril-specific and requires expert knowledge—for example in seismology, meteorology, or hydrology. Hence, the only heterogeneous, silo-type, parts of the CAT modeling pipeline are all the steps leading to the assessment of the hazard intensity footprint I and of its rate λ. While the most common perils in CAT models [10,11,12] remain earthquakes [13,14], tropical and extratropical cyclones [15,16], and floods [17,18], others have been progressively added, such as convective storms with hail and tornados [19,20], cyber-attacks [21,22], epidemics [23,24], terrorist attacks [25,26], and wildfires [27]. Secondary perils, also included in some CAT models, include fire-following earthquakes [28], storm surges [29], and business interruption [30]. Other perils, primary or secondary, also recently considered in the CAT modeling framework include landslides, tsunamis, and volcanic eruptions [4,12]. Others have yet to be fully integrated in the CAT modeling paradigm, such as heatwaves [31], crop failures [32], and asteroid impacts [33,34]. Social unrest and war, to only cite a few additional perils, have yet to be implemented to the best of the author’s knowledge, although computational methods are already available [35,36]. A peril is often not implemented if not yet demonstrated to be insurable, which is most commonly due to a lack of data or models to assess risk and set premiums [26]. It has also been argued that some man-made risks are not calculable [37] or simply too costly to insure.

For each peril considered, a different set of terms, concepts, and models is employed. This hampers comparisons and makes the implementation of any new peril in the CAT modeling framework a challenge. To improve comparability and find equivalences in physically different hazard processes, this article reviews the different steps involved in probabilistic hazard assessment for more than 20 perils and categorizes them in terms of event source (Section 2.2), event size distribution (Section 2.3), and hazard intensity footprint (Section 2.4). Finally, the hazard process is harmonized in terms of energy transfer since it is applicable to any peril (Section 3). This review serves several purposes. Pedagogical and encyclopedic in nature, it first illustrates the richness of the space of potential hazards faced by society. Second, by treating and quantifying all perils and their hazards in a common way, it provides the basic knowledge needed to develop CAT modeling as an independent scientific discipline. Finally, this review can be viewed as a preliminary step to developing multi-risk models that would consider all the interconnections between the natural, technological, and socio-economic systems, within the context of CAT accumulation risk.

## 2. Review of Hazard Modeling Parameterization per Peril

We reviewed the literature on CAT modeling, as well as other works which can provide inputs to CAT models. We attempted to cover as many perils as possible for the three hazard steps (Section 2.2, Section 2.3 and Section 2.4). Note that we distinguish *peril*, a potential cause of damage, from *hazard*, the danger arising from a peril [4]. In other words, a peril can be an earthquake, a storm, or an industrial explosion, while their matching hazards (in terms of intensity) are ground shaking, strong wind, and blast overpressure, respectively. The term *event* then represents one instance from a given peril (one earthquake, one storm, etc.). To make this review tractable, we only consider first-order processes and mention seminal studies and reviews for the reader to explore each peril in more detail. For each step of the probabilistic hazard assessment procedure, peril characteristics are listed in alphabetical order. Some perils can display different hazardous phenomena, in which case each phenomenon is associated to a *sub-peril*. Not all perils are described systematically in Section 2.2, Section 2.3 and Section 2.4. Note that the proposed categorization is aimed at facilitating peril comparison, not at forcing them into strict boxes. For convenience, Table A1 of Appendix A provides a list of the variables and parameters mentioned in this review.

### 2.1. Data

To illustrate some of the possible models (mainly for source-type description and size distribution parameterization—see Section 2.2 and Section 2.3), the following databases were used:

- **Asteroid impacts (fireballs):** The Fireballs Reported by US Government Sensors [38] dataset for the period 15 April 1988–21 August 2022, available online: https://cneos.jpl.nasa.gov/fireballs/ (accessed on 31 August 2022).
- **Blackouts:** Dataset of numbers of customers affected in electrical blackouts in the United States between 1984 and 2002 [39], available online: https://aaronclauset.github.io/powerlaws/data/blackouts.txt (accessed on 31 August 2022).
- **Cyber-attacks:** The 2005–2018 Privacy Rights Clearinghouse (PRC) catalogue [40] for category hacking/malware, available online: https://privacyrights.org/data-breaches (accessed on 31 August 2022).
- **Earthquakes:** The 1900–2012 International Seismological Centre-Global Earthquake Model (ISC-GEM) Global Instrumental Earthquake Catalogue [41], available online: http://www.isc.ac.uk/iscgem/ (accessed on 31 August 2022); the fault source model of the 2013 European Seismic Hazard Model (ESHM13) [42], available online: http://hazard.efehr.org/en/Documentation/specific-hazard-models/europe/overview/active-faults/ (accessed on 31 August 2022).
- **Epidemics:** The Global Epidemics Dataset [43], available online: https://zenodo.org/record/4626111 (accessed on 31 August 2022).
- **Heatwaves:** Temperature data for July 2022 in France from the Météo-France data portal [44], available online: https://donneespubliques.meteofrance.fr/donnees_libres/Txt/Synop/Archive/synop.202207.csv.gz (accessed on 31 August 2022).
- **Landslides:** Inventory of events triggered by the 2008 Wenchuan, China, earthquake, courtesy of Dr. G. Li and Prof. J. West [45].
- **River flooding:** Dataset of flood peaks at the Potomac River between 1895 and 1986, provided in Table 1 of [46], a textbook example.
- **Terrorism:** Dataset of the severity of terrorist attacks worldwide from 1968 to 2006, measured as the number of directly resulting deaths [39,47], available online: https://aaronclauset.github.io/powerlaws/data/terrorism.txt (accessed on 31 August 2022).
- **Tropical (and extra-tropical) cyclones**: The International Best Track Archive for Climate Stewardship (IBTrACS) [48], available online: https://www.ncei.noaa.gov/products/international-best-track-archive?name=ibtracs-data (accessed on 31 August 2022).
- **Tsunamis**: The NCEI/WDS Global Historical Tsunami Database [49], here for the selected period 1900–2022, available online: https://www.ngdc.noaa.gov/hazard/tsu_db.shtml (accessed on 31 August 2022).
- **Volcanic eruptions:** The global database on large magnitude explosive volcanic eruptions (LaMEVE) [50], available online: https://www2.bgs.ac.uk/vogripa/view/controller.cfc?method=lameve (accessed on 31 August 2022).
- **Wildfires:** The FRY global database of fire patches [51], available online: https://data.oreme.org/doi/view/0e999ffc-e220-41ac-ac85-76e92ecd0320#FRY (accessed on 31 August 2022).

While most datasets are global, the others go from regional to local. Different time periods are also represented. These spatiotemporal heterogeneities have, however, no significant impact on the present review since the data are mainly used for illustration purposes with no quantitative comparison being provided. Moreover, most datasets are annualized. A color scheme has been developed to distinguish between different peril categories in the next figures. The scheme is given in Table A2 of Appendix A.

### 2.2. Event Source and Event Size

The size S of an event is constrained by the source from which it originates. A *source* is here defined as the energy stock that drives the event. Each source represents a unique *object* with peril-specific characteristics. For instance, a tropical cyclone manifests itself from an atmospheric low-pressure system that moves along a track, while an earthquake corresponds to the rupture of a fault plane under tectonic loading—two very different objects. We define five categories of sources: *point sources* (Section 2.2.1), *line sources* (Section 2.2.2), *area sources* (Section 2.2.3), *track sources* (Section 2.2.4), and *diffuse sources* (Section 2.2.5). For each peril, we use the most common size metrics and, of those, preferentially the one most closely related to energy (see Section 3). We only list one model per peril to estimate the event size as a function of the source parameters, based on first principles and other simple empirical relationships. References to more sophisticated models are given for completeness.

#### 2.2.1. Point Source

The point source of coordinates $\left(x_{0},y_{0}\right)$ is the simplest source type. The event size depends on the energy stock implicitly encoded in that point.

**Figure 2:**
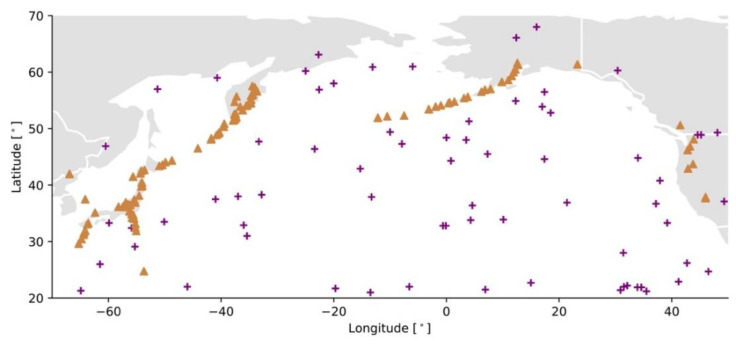
Examples of point sources (asteroid impact and volcanic eruption cases) in the North Pacific region. Fireball locations (plus signs) in the dataset of Fireballs Reported by US Government Sensors [38]. Active volcanoes (triangles) represented by the eruptions that occurred in the past 10,000 years in the LaMEVE database [50].

- **Asteroid (or comet) impacts:** The source is the impact site, which is random and uniform in space (Figure 2). The stored energy is defined by the characteristics of the impactor and the event size is directly expressed in terms of kinetic energy E [J],
  (2) $$E=\frac{1}{2}mv^{2},$$
  with m [kg] the mass of the body and v [m/s] its velocity. The typical characteristics of the impactor are a density of ρ≈ 3 g/cm^3^ (stony asteroid), 8 g/cm^3^ (iron asteroid), or 0.5 g/cm^3^ (comet) and a velocity of v≈ 20 km/s (asteroid) or 50 km/s (comet) [52]. Equation (2) is an oversimplification of the process and does not consider atmospheric deceleration, disruption, or ablation processes, nor ground penetration [34,52,53].
- **Explosions (accidental):** The source is a container of explosive material. Sources of severe accidental explosions are located at industrial sites, so-called Seveso sites. The size of the event is defined by the blast energy E [kJ], which is a function of the mass and chemical characteristics of the explosive substance. It is usually described in TNT mass equivalent $m_{TNT}$ [kg]. For a vapor cloud explosion (VCE), or fuel-air explosion, we have
  (3) $$m_{TNT}=\frac{E}{\Delta H_{c\left(TNT\right)}}=\frac{\eta m_{v}\Delta H_{c\left(gas\right)}}{\Delta H_{c\left(TNT\right)}},$$
  where η is the explosion efficiency, or fraction of available combustion energy participating in blast wave generation, $\Delta H_{c\left(gas\right)}$ [kJ/kg] is the heat of combustion of the material, and $m_{v}$ [kg] is the mass of flammable vapor release, itself a percentage of the total mass of hazardous material. $\Delta H_{c\left(TNT\right)}\approx$ 4200 kJ/kg is the heat of combustion of TNT. For an explosion at a fuel storage site or refinery, $\Delta H_{c\left(gasoline\right)}\approx$ 46.4 MJ/kg and η=0.1 [54,55] can be used as an example. Another type of explosion is the boiling liquid-expanding vapor explosion (BLEVE), resulting from the sudden vaporization of a liquid [56]. Related perils include fire and toxic material release [57,58].
- **Explosions (armed conflicts, terrorism):** The source is a bomb, whose size is known by design. For conventional blasts, Equation (3) can be used with high explosives considered as source material (e.g., TNT). For non-conventional blasts, such as a nuclear explosion, a simple equation of the yield is
  (4) $$E\sim \frac{m_{tot}}{8}{\left(\frac{\Delta R\cdot \log n}{\tau }\right)}^{2},$$
  where $m_{tot}$ is the total mass of the spherical bomb (core + tamper), ΔR is the difference between the expanded radius and the initial radius of the sphere, τ is the time required for a neutron born in a fission to subsequently strike and fission another nucleus, and n is the number of neutrons liberated per fission (n=2.637 for Uranium 235 and n=3.172 for Plutonium 239) [59]. In contrast to accidental explosions, sources of intended explosions are mobile and correlate with population density and specific (critical) infrastructure targets [26]. It can be considered a sub-peril of armed conflicts and terrorist attacks, which have a diffuse source (see Section 2.2.5). A related peril is radiation in the case of a nuclear attack.
- **River floods:** The source is a river system associated to a catchment basin. It can, however, be represented by (or concentrated at) a point source characterized by the peak discharge $Q_{p}$ [m^3^/s] at a point of the river. For a small basin (≈1 km^2^), it is estimated with the Rational Formula
  (5) $$Q_{p}=\eta \frac{h_{c}}{t_{c}}A,$$
  with η the runoff coefficient (which depends on soil conditions and surface characteristics, such as asphalt versus grass), $h_{c}$ [m] the critical rainfall, $t_{c}$ the concentration time [s] (function of flow distance and terrain slope), and A the catchment area [m^2^] [60,61]. If $t_{c}$ is defined as the duration of the rainfall event, $h_{c}/t_{c}$ then represents the rainfall intensity with the role of slope included in η. For greater basins, non-linearities must be included, such as the topography and the non-stationary flow observed on hydrographs [60]. An empirical relationship approximating the process can otherwise be used with
  (6) $$E\left[Q_{p}\right]=c_{1}A^{c_{2}}h^{c_{3}}\phi^{c_{4}},$$
  where $E\left[Q_{p}\right]$ is the expected annual peak discharge [m^3^/s], A [km^2^] the catchment area, h the annual average rainfall in the catchment [mm], ϕ the upstream catchment slope [m/km], and $c_{1}$ to $c_{4}$ empirical parameters [62].
- **Volcanic eruptions:** An active volcano transfers heat and matter from the Earth’s interior to outside the volcanic edifice. Most eruptions occur along the Ring of Fire (Figure 2). The event size is the volume of matter ejected V [km^3^], which is also the main parameter of the Volcanic Explosivity Index (VEI) [63]. Other characteristics of the magma, such as temperature T, allow the thermal energy released during the eruption to be estimated [64] (see Section 3).

#### 2.2.2. Line Source

The line source is composed of n segments with coordinates $\left(\left(x_{1},\cdots ,x_{n+1}\right),\left(y_{1},\cdots ,y_{n+1}\right)\right)$. For a “line source” with a time stamp, see Section 2.2.4 on track sources. Various source characteristics can be associated to each point or segment.

**Figure 3:**
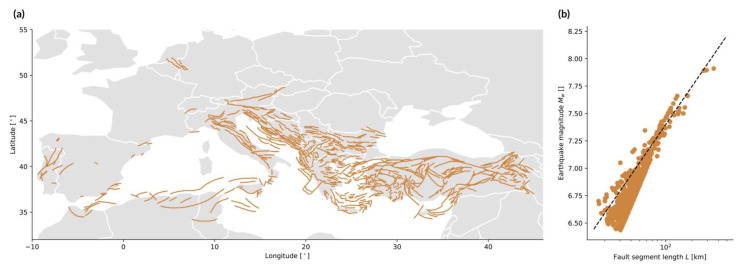
Example of line sources (earthquake case). (**a**) Fault segments in the 2013 European Seismic Hazard Model (ESHM13) [42]; (**b**) mean magnitude $M_{w}$ versus effective fault segment length l in the ESHM13, compared to Equation (8) (dashed line) with $c_{1}=5.08$ and $c_{2}=1.16$ [66].

- **Earthquakes:** The source is a fault, i.e., a planar rock fracture which shows evidence of relative movement. There is often no need to explicitly define an area source as the line carries dip and width information (Figure 3a). The seismic energy released by an earthquake is proportional to the seismic moment $M_{0}=\mu Au$ [N·m], with $\mu =3.3\times 10^{10}$ Pa the rock shear modulus, A [m^2^] the rupture surface area, and u [m] the average displacement on the fault. The earthquake’s size is, however, commonly described in terms of moment magnitude [65]
  (7) $$M_{w}=\frac{2}{3}\log_{10}\left(M_{0}\cdot 10^{7}\right)-10.7,$$
  Usually, empirical relationships linking magnitude and fault geometry are used instead of the other physical characteristics of the rupture, such as
  (8) $$M_{w}=c_{1}+c_{2}\log_{10}l,$$
  with l the surface rupture length and $c_{1}$ and $c_{2}$ fitting parameters as a function of the fault mechanism (normal, reverse, or strike-slip) [66] (Figure 3b).
- **Storm surges:** The source is a storm, and more precisely the low-pressure region above the water mass combined with strong winds. It may be considered a line source since the event size is defined in terms of the water height h along the coastline [29]. The storm surge can be related to storm maximum windspeed $v_{max}$, for example with a polynomial function of the form
  (9) $$h=c_{1}v_{max}+c_{2}v_{max}^{2}+c_{3}v_{max}^{3},$$
  where the empirical parameters $c_{1}$, $c_{2}$, and $c_{3}$ are site-specific [67]. Although the match between hurricane windspeed and storm surge height ranges is given on the Saffir–Simpson Hurricane Wind Scale, the $v_{max}$ indicator is often not sufficient for estimating the actual storm surge [68].
- **Tornados:** The simplified source of a tornado track is a line with no intensity variability along its length [20] (otherwise it is modelled as a track source—Section 2.2.4). The event size is defined in terms of maximum wind speed $v_{max}$, which is the main parameter of the Enhanced Fujita Scale [69]. Additional parameters of the source, such as location, length, and width (or maximum radius), are sampled from historical data [70].
- **Tsunamis (triggered by an earthquake):** In this case, the source is an underwater fault line. The size of the event is commonly defined by both wave velocity and wave height at arrival on the coast, which is equivalent to the hazard intensity footprint. This applies also to tsunamis generated by other non-meteorological sources, including asteroid impacts, landslides, and volcanic eruptions. For an earthquake trigger, the initial size of the tsunami above the rupture can be estimated in terms of potential energy E [J] (following the box-shaped ‘waterberg’ method) by
  (10) $$E=\frac{1}{2}\rho_{w}glwu^{2},$$
  with $\rho_{w}=1000$ kg/m^3^ the water density, g the gravitational acceleration, l the earthquake rupture length [m], w the wavelength or width of the area displaced [m], and u the upward rupture displacement [m] [71]. For other formulations, see [72].

#### 2.2.3. Area Source

An area source is a localized surface large enough that it cannot be simplified by a point source (Section 2.2.1) and small enough that it is not a diffuse environmental source (Section 2.2.5). There is no strict definition of an area source. It can be characterized by a geometrical shape, such as a rectangle or an ellipse, or by an irregular patch defined by the coordinates of its contour. All physical characteristics are assumed to be spatially homogeneous within the source. An area source, such as a large warehouse stocked with explosive material, is commonly simplified by a point source [73]. An area source may represent a region in which the peril process is kept hidden, such as a territorial division in a compartmental epidemic model (with integration method). However, the heterogeneity of the real-world environment makes the use of a diffuse source more realistic in many cases (e.g., landslides—see Section 2.2.5). Finally, an area source can also represent uncertainty on a fault line [42], while a line source may suffice to characterize an area (e.g., an earthquake rupture plane or tornado path, as previously shown). Perils for which an area source is most common are:

- **Hail:** The source is a convective storm, with hail as a sub-peril alongside strong winds, tornados, lightning, and heavy rain. It is described by the area in which hailstones are found, with the size of the event defined in terms of the maximum hailstone diameter l [cm] [19]. Hail cells have been approximated by so-called storm boxes [19] or ellipses [74]. Their location, size, and shape are constrained by meteorological observations [74]. The temporal evolution during an event can also be considered, in which case a track source should be used [74]. Note that it is a case where event source and hazard footprint cover the same area (see Section 2.4.2).
- **Urban fires and wildfires:** Fires in both wildland and urban areas were originally modelled as ellipses, with the fire spread rate R [m/min] defined as
  (11) $$R=R_{null}\left(1+\left|\phi_{s}+\phi_{w}\right|\right)\frac{1-\varepsilon }{1-\varepsilon \cos\phi },$$
  with $R_{null}$ the spread rate on flat terrain and without wind as a function of the combustible characteristics (i.e., the source), $\phi_{s}$ the direction of maximum slope, $\phi_{w}$ the wind direction, ε the eccentricity function of windspeed and terrain slope, and ϕ an arbitrary direction [75,76]. Despite its simplicity, Equation (11) produces reasonable estimates of fire spread in a uniform environment. It is now more common to use cellular automata to model fires by considering diffuse sources instead [28] (see Section 2.2.5 and Section 2.4.3). Yet, ellipses can be used to define the seed events from which greater fires can propagate [76].

#### 2.2.4. Track Source

**Figure 4:**
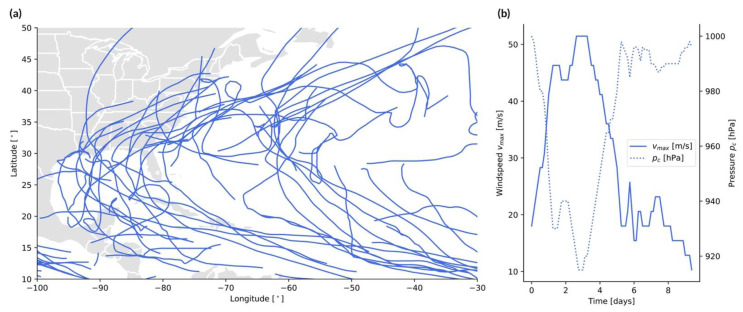
Example of track sources (tropical cyclone case). (**a**) Tracks of recent Atlantic hurricanes (2020–2022) in the IBTrACS database [48]; (**b**) windspeed $v_{max}$ versus central pressure $p_{c}$ along the track of 1992’s Typhoon Yvette, based on IBTrACS data as shown in [48].

The track source is the combination of a point source and of a line source, with the event footprint defined at any given time from a point along the line, i.e., the track $\left(\left(x_{1},\cdots ,x_{n}\right),\left(y_{1},\cdots ,y_{n}\right),\left(t_{1},\cdots ,t_{n}\right)\right)$. This applies to storms, such as windstorms, tropical cyclones, and other related perils (note that the point source could be replaced by an area source, for example in the case of hail). Examples of hurricane tracks are shown in Figure 4a.

**Figure 5:**
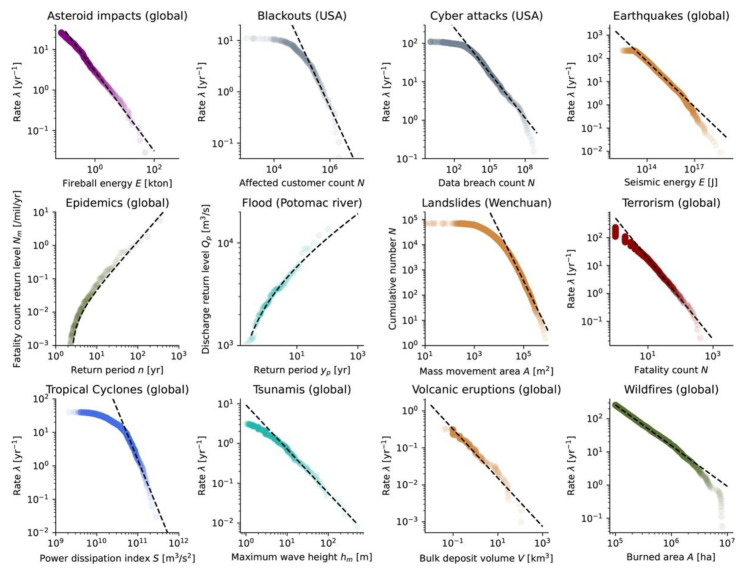
Size distribution of various perils, fitted by a simple power-law (most perils), a GEV distribution (river flooding), or a GPD (epidemics). Note that the results are sensitive to the choice of minimum size threshold, with fitted parameters to be used carefully when extrapolating. Data sources (Section 2.1): asteroid impacts [38], blackouts [39], cyber-attacks [40], earthquakes [41] (with $\log_{10}E=5.24+1.44M$ [117]), epidemics [43], landslides [45], flooding [46], terrorism [39], tropical cyclones [48], tsunamis [49], volcanic eruptions [50], and wildfires [51]. See the text within Section 2.3 for the values of the fitting parameters.

- **Tropical cyclones:** The source is an area of low pressure over a large water surface, which moves along a track over time t. The genesis point, trajectory, and end point of the storm are stochastic and derived from past observations [77]. The event size at any given time is defined by the maximum wind speed
  (12) $$v_{max}\left(t\right)=\sqrt{\frac{B\left[p_{n}-p_{c}\left(t\right)\right]}{\rho e}},$$
  with $p_{c}$ [Pa] the cyclone’s central pressure, $p_{n}$ [Pa] the ambient pressure outside the cyclone, ρ= 1.15 kg/m^3^ the air density, 1≤B≤2.5 the Holland B parameter, and e Euler’s number [78]. Notice the anticorrelation between $v_{max}\left(t\right)$ and $p_{c}\left(t\right)$ in Figure 4b, in agreement with Equation (12). The windspeed can then be used to estimate the event size on the Saffir–Simpson Hurricane Wind Scale. Along the track, the windspeed progressively increases, as the tropical cyclone grows, and then decreases, as the storm makes landfall, weakens, and dies off (Figure 4b). One can define the size of an individual storm in terms of total power dissipation by integrating over the wind profile along the entire track [79] (see Figure 5 and Section 3).

#### 2.2.5. Diffuse Source

The diffuse source is the part of the environment—natural, technological, or socio-economic—which acts as a spatially extended energy stock. The environment, or system, can be defined on an $n_{x}\times n_{y}$ geographical grid with coordinates $\left(\left(x_{1,1},\cdots ,x_{n_{x},n_{y}}\right),\left(y_{1,1},\cdots ,y_{n_{x},n_{y}}\right)\right)$. It may also be represented as a graph composed of n nodes with spatial coordinates $\left(\left(x_{1},\cdots ,x_{n}\right),\left(y_{1},\cdots ,y_{n}\right)\right)$ and links defined in an adjacency matrix (as, for example, in the case of connected infrastructures). It could finally be a set of n moving agents with coordinates $\left(\left(x_{1},y_{1})(t\right),\cdots ,\left(x_{n},y_{n})(t\right)\right)$. Each element of the diffuse source has additional characteristics necessary for the estimation of event size. In contrast to other source types, the event size is not well constrained without dynamical modeling, except for the maximum possible size maxS often being the size of the system itself. This section describes the source of a *seed event*, which can be a point or an area which does not characterize the event in its entirety. The event size S depends on how the event grows, which is described in Section 2.4.3 on hazard footprint modeling. In some of those cases, event size and footprint intensity can be indistinguishable and described by the same metric.

- **Armed conflicts (incl. terrorism):** The source is a hierarchical group of individuals, ranging from small terrorist organizations to large (trans)national armies. The size of the seed event is constrained by the funds and people power at the disposal of the attacker, as well as by the group’s network structure and utility function. The process is highly dynamic, as the various agents are mobile and opponents can allocate resources to defend against an attack [25,26]. The size of an event depends directly on the type and number of weapons. It can be a group of fighters (with firearms or non-firearms—see Section 2.4.3), conventional weapons (expressed as a TNT-equivalent, e.g., Equation (3)), or non-conventional weapons, including chemical, biological (see epidemic), radiological, nuclear (e.g., Equation (4)), and cyber- attacks. Those weapon types, which require different hazard modeling strategies, can be considered different sub-perils of an armed conflict. The event size S is commonly defined in terms of the fatality count N summed over all attacks taking place during the conflict (N then directly represents the human loss L in the risk component of the CAT model).
- **Blackouts:** The source of a blackout is a current overload due to a local disturbance in the power grid. This system is composed of generator nodes (i.e., power plants), transmission nodes, and distribution nodes connected via transmission lines. A seed event can correspond to the tripping of several lines due to tree contact for example, which can be caused by lack of tree trimming or by a storm [80]. The event is only called a blackout if a relatively large number of consumers N is affected by the loss of electricity. The event size can also be defined in terms of unsupplied energy E [MWh]. The event size depends on how the overload propagates through the power grid through cascading failures.
- **Business interruptions:** The source is any business that is shut down due to direct damage by some natural or man-made event. Although a business location can be represented by a point source or extended area source, a catastrophic event consists of the aggregation of disruptions at many locations in the built environment, which may include a supply chain network in the case of contingent business interruption. The event size is directly defined in terms of revenue loss [30].
- **Crop failures (due to pest):** The source is a pest, such as an insect, a virus, a grazing animal, or some other invasive species that damages the crops. The size of an event depends on the complex interactions between the pest and crop growth within the crop production system where natural predators and/or pesticides may also participate [32]. Note that crop failure can also be due to climatic stress, represented by extreme temperature changes, droughts, as well as meteorological (hail), hydrological (flood), and ecological (field fire) events. In those cases, crops only represent the exposure layer of the CAT model. The event size is commonly defined in economic terms, such as farming production yield loss. However, it could, in theory, be defined by pest biomass before any consideration of crop damage.
- **Cyber-attacks:** The source of a cyber-attack is a malicious agent (a hacker) acting for personal gain or on behalf of a governing entity. Cyber-attacks can include theft of data or currency, ransoms, business interruption, or some other forms of system destabilization. The attack occurs, by definition, via electronic communication networks and virtual reality [21]. One particularity of cyber-attacks is that they are not geographically bound. They can cascade into greater events [22] via highly dynamic processes [21]. Their size is defined in terms of the number of data breaches N in the common case of data exfiltration. However, this depends on how the initial attack propagates through the IT system. Since N is often the number of actual breaches and not of attempted breaches, the event size directly reflects the loss in the risk domain after considering the level of vulnerability of the exposed system. Hazard and risk are intertwined since both the type and size of a cyber-attack depend on the attacked system. For example, a cyber-heist on a banking system is different from a distributed denial of service (DDoS) attack (with S here expressed in gigabits per second [Gbps]), itself different from a cyber-attack on a power grid or other connected critical infrastructure. Many other types of events exist which could go as far as a cyber-war [22].
- **Epidemics:** The source of an epidemic is the first infection in the human population. This requires the pathogen and susceptible hosts to be in contact in adequate numbers. The size of an event can be the number of infections N, which depends on how the epidemic propagates, as a function of the basic reproduction number
  (13) $$R_{0}=\beta N_{tot}\Delta t,$$
  where β is the transmission parameter, Δt the recovery delay, and $N_{tot}$ the total population. The parameters β and Δt depend on the vector, which can be a virus, bacterium, fungus, or parasite [24]. When an epidemic spreads to multiple continents, it becomes a pandemic. The total number of fatalities, which is the number of infections times the mortality rate, is the final loss. However, it may also be considered as the event size since the mortality rate also depends on the pathogen, and not only on the human vulnerability (function of age, gender, and health condition).
- **Landslides:** The source is the set of terrain patches with an unstable slope ϕ, which is controlled by topographic and soil characteristics. The size of the seed event can be the area A [km^2^] or volume V [km^3^] of each patch or set of patches. The area that is unstable is defined by a Factor of Safety ($F_{s}$) lower than 1, since it is the ratio of resisting forces to driving forces. A simple formulation is
  (14) $$F_{s}=\frac{\frac{C}{\rho_{s}gh}+\cos\phi \left(1-\eta \frac{\rho_{w}}{\rho_{s}}\right)\tan\phi_{int}}{\sin\phi },$$
  where h [m] is the depth perpendicular to surface (or thickness) of the soil, g is the gravitational acceleration, C [N/m^2^] is the soil cohesion, $\rho_{s}$ [kg/m^3^] is the soil density, $\rho_{w}=$ 1000 kg/m^3^ is the unit weight of water, ϕ is the slope angle, $\phi_{int}$ is the internal friction angle of the soil, and $\eta =h_{w}/h$ is the relative wetness representing the ratio between water column height $h_{w}$ and soil height h. For additional formulations, see [81,82]. An increase of η due to heavy rain further decreases $F_{s}$, making previously stable slopes unstable [82]. $F_{s}$ is also involved in landslide triggering by earthquake ground shaking [83].
- **Social unrest:** The source of social unrest is the part of the population which has a high level of grievance against the governing entity [35]. The first individuals turning violent, who can be anyone in the system, can lead to a riot, i.e., an aggregate act of violence against individuals and property, which includes looting and setting fires as sub-perils [84]. The event can, however, be avoided if enough security is at the disposal of the government [35]. The dynamics is reminiscent of what can occur during an armed conflict (see above), with an extreme social unrest event potentially turning into a revolution. The event size could, in theory, be defined in terms of the number of rioters N.
- **Urban fires (accidental or malicious):** Fire can be considered a sub-peril of industrial accidents [58], armed conflicts [85], and social unrest [84], as well as a secondary peril of earthquakes [28]. The source is some combustible material that is set alight. The event size, defined in terms of burnt area A, depends on how the fire propagates in the environment, as in the case of a wildfire (see below). If an elliptical event is realistic in a uniform environment (Equation (11)), it is not in most real-world situations.
- **Wildfires:** The source of a wildfire has two components: a trigger for ignition and some combustible material (i.e., vegetation). The main cause of wildfires globally is anthropogenic, with fires started intentionally or accidentally. This ranges from power line ignition to arson via a forgotten cigarette butt [86]. Lightning strikes are the most important natural ignition trigger for wildfires [87]. In this case, the occurrence of a seed event depends on the continental lightning rate [flashes/min]
  (15) $$\lambda_{flash}=c\cdot 3.44\cdot 10^{-5}\cdot h^{4.9},$$
  A function of the convective cloud top height h [km] and a resolution- and model-dependent scaling factor c [87,88]. The size of the event, described in terms of burnt area A, depends on the propagation process, a function of the characteristics of the environment, such as terrain, fuel, and meteorological conditions (see Equation (11)). Conditions are more favorable for a wildfire during a drought [89]. In the CAT modeling context, losses occur in the wildland–urban interface, defined as an area covered by more than 50% vegetation with more than one housing unit per 1.62 ha [27]. An ignition index can be calculated to map the potential size of an event as a function of dead fuel moisture, temperature, and vegetation species flammability among other parameters [90].

### 2.3. Event Size Distribution

The probabilistic nature of a hazard is described by the rate of events $\lambda_{i}$ as a function of the event size $S_{i}$ (or by the return period $\Delta T_{i}=1/\lambda_{i}$). There are two main statistical approaches, commonly the *power-law distribution* [39,91,92], for discrete events, and the *Generalized Extreme Value* (GEV) *distribution* and *Generalized Pareto distribution* (GPD) [93], for events derived from continuous flows. Figure 5 shows the size distribution of the perils from Section 2.1 that can be fitted by a power-law, GEV, or GPD. Any peril can, however, be described by various statistical distributions, with the choice often depending on the discipline’s most accepted approach for event definition and event count [94,95,96]. We here assume the independence of events as well as the stationarity of event occurrences over time.

#### 2.3.1. Power-Law Distribution

The complementary cumulative distribution function (CCDF) of a power-law is

(16) $$\mathrm{Pr}\left(S\right)=\int_{S}^{+\infty }\mathrm{pr}\left(u\right)du={\left(\frac{S}{S_{min}}\right)}^{-\left(\alpha -1\right)},$$

with S the event size, $S_{min}$ the minimum event size threshold, and α the power exponent [39]. The size distribution of a hazard is most often described by the annual rate

(17) $$\lambda \left(\geq S\right)=\lambda \left(\geq S_{min}\right)\mathrm{Pr}\left(S\right)=\lambda \left(\geq S_{min}\right){\left(\frac{S}{S_{min}}\right)}^{-\left(\alpha -1\right)},$$

generally expressed in the following empirical form

(18) $$\log_{10}\lambda \left(\geq S\right)=a-b\log_{10}S,$$

with $a=\log_{10}\left(\lambda \left(\geq S_{min}\right)S_{min}^{\alpha -1}\right)$ and b=α−1 the slope of the size distribution in a log–log plot (also called the shape parameter of the classical Pareto distribution). We will consistently use b (i.e., cumulative form) in the following review.

- **Armed conflicts (incl. terrorism):** The size distribution follows Equation (18), with S=N as the number of fatalities [97]. In Ref. [97], a value of b≈1 was obtained for various types of conflicts (war, banditry, gang warfare). In Ref. [98], a value of b≈0.5 was obtained for interstate wars taking place between 1820 and 1997 and the 1465–1965 European great power wars. In Ref. [39], a value of b=0.7 was calculated for wars between 1816 and 1980. In the case of terrorism worldwide from 1968 to 2006, we obtain a=2.714 and b=1.45 (Figure 5), close to the value of b=1.4 found by [39,47] for the same dataset.
- **Asteroid impacts:** The flux of small near-Earth objects colliding with our planet follows a power-law in the form of Equation (18), with S=E [kton] as the energy and a= 0.5677 and b= 0.90 globally [99]. In Ref. [92], a value of b=1.02 was obtained when including more recent data. Considering data up to 2022, we obtained a= 0.468 and b= 0.99 (Figure 5).
- **Blackouts:** The size distribution follows Equation (18), with S=N as the number of customers affected. In Ref. [100], the range 0.5≤b≤1.0 was observed for different countries. For data from the United States, Ref. [39] obtained b=1.3, while we obtained a=6.812 and b=1.18 (Figure 5) for the same dataset.
- **Cyber-attacks:** The size distribution follows Equation (18), with S=N the number of personal identity losses or data breach volume (used as the example in this case). In Ref. [101], a value of b=0.7 was obtained when using data from the Open Security Foundation for the 2000–2008 period. Considering hacking events from the public dataset published by the Privacy Rights Clearinghouse [40], we obtained a=3.184 and b=0.4 for the 2005–2018 period.
- **Earthquakes:** Although the size distribution of earthquakes also follows a power-law in the seismic energy domain with S=E (Equation (18), as with the a=11.004 and b=0.65 values shown in Figure 5, and with $\log_{10}E\propto 1.5M$) [102], the Gutenberg–Richter (exponential) law is used in virtually all cases [103], as a function of the magnitude M. It yields a Gutenberg–Richter slope of 1.5b=0.98 globally [41] which is close to unity, known as the standard value for tectonic earthquakes.
- **Landslides:** The size distribution follows Equation (18), with S=A [km^2^] the landslide area or S=V [km^3^] the landslide volume. Conversion from area to volume can be performed with the empirical scaling relationship $V=cA^{1.5}$ [104]. For S=A, a review of more than 20 analyses provides b=1.3±0.6 [105]. For landslides triggered by the 2008 Wenchuan earthquake for instance [45], we find b=1.99 at the tail of the distribution (Figure 5), which is in agreement with [106] who obtained b=2.07.
- **Tsunamis:** The size distribution follows Equation (18), with $S=h_{max}$ the maximum wave height (i.e., tsunami runup). Ref. [107] obtained 0.8≤b≤1.3 for different locations along Japan. For global data [49], we find a=0.967 and b=1.11 (Figure 5).
- **Volcanic eruptions:** The size distribution follows Equation (18), with S=V [km^3^] as the erupted volume. Considering all volcanic eruptions which occurred after the year 1000 in the LaMEVE database [50], we obtain a= −1.156 and b= 0.66 (Figure 5). A recent review of large VEI eruptions indicates that VEI-7 events recur every 500–1000 years [108]. Our parameters lead to 300–1300 years for the V range of VEI-7 events.
- **Wildfires:** The size distribution follows Equation (18), with S=A [km^2^] as the size of the wildfire as defined by the burned area A. Ref. [109] reviewed the literature and mentioned 1.1≤b≤1.8 for China and the United States. Ref. [39] calculated b=1.2 for U.S. federal land. Ref. [92] found b=0.82 for fires in Angola and b=1.28 for fires in Canada. For the FRY catalogue [51], we obtained a= 8.553 and b= 1.23 (Figure 5).

#### 2.3.2. Generalized Extreme Value (GEV) Distribution

Events such as storms and floods, which originate from continuous flows (wind and water discharge, respectively), must be defined as extreme instances. To define an event, a threshold or maximum estimate is then considered, following the rules of Extreme Value Theory [93]. When considering the maximum value $X_{max}$ over a fixed period (i.e., the block maxima approach), the Generalized Extreme Value (GEV) family of distributions applies which, in CDF form, is

(19) $$\mathrm{Pr}\left(X_{max}\leq S\right)=\{\begin{array}{cc} \exp\left[-{\left\{1+\xi \left(\frac{S-\mu }{\sigma }\right)\right\}}^{-\frac{1}{\xi }}\right] & \mathrm{for}\;\xi \neq 0\\ \exp\left[-\exp\left\{-\left(\frac{S-\mu }{\sigma }\right)\right\}\right] & \mathrm{for}\;\xi =0 \end{array},$$

with the “size” S termed the return level (“$z_{p}$”) in GEV parlance, μ the location parameter, σ the scale parameter, and ξ the shape parameter. ξ>0 corresponds to the Fréchet distribution, ξ<0 to the Weibull distribution, and ξ=0 to the Gumbel distribution [93]. Considering the probability of exceedance instead,

(20) $$\mathrm{Pr}\left(X_{max}>S\right)=1-\mathrm{Pr}\left(X_{max}\leq S\right)=\frac{!}{\Delta T},$$

where ΔT is the return period. Inverting Equation (20) gives the so-called *return level plot*

(21) $$S=\{\begin{array}{cc} \mu -\frac{\sigma }{\xi }\left[1-{\left\{-\log\left(1-\frac{1}{\Delta T}\right)\right\}}^{-\xi }\right] & \mathrm{for}\;\xi \neq 0\\ \mu -\sigma \log\left\{-\log\left(1-\frac{1}{\Delta T}\right)\right\} & \mathrm{for}\;\xi =0 \end{array},$$

Introducing $y_{p}=-1/\log\left(1-1/\Delta T\right)$ (i.e., a return period) in Equation (21) yields [110]

(22) $$S=\{\begin{array}{cc} \mu +\frac{\sigma }{\xi }\left[y_{p}^{\xi }-1\right] & \mathrm{for}\;\xi \neq 0\\ \mu +\sigma \log y_{p} & \mathrm{for}\;\xi =0 \end{array},$$

(see flood case below).

An alternative representation of extremes that uses more of the available data consists of analyzing excesses over a high threshold (the so-called Peak-Over-Threshold, POT, method). In this case, the Generalized Pareto distribution (GPD), a type of power-law [91], applies. Its CCDF takes the form

(23) $$\mathrm{Pr}\left(X>S|X\rangle \mu \right)=\{\begin{array}{cc} {\left[1+\xi \left(\frac{S-\mu }{\sigma }\right)\right]}^{-\frac{1}{\xi }} & \mathrm{for}\;\xi \neq 0\\ \exp\left[-\frac{S-\mu }{\sigma }\right] & \mathrm{for}\;\xi =0 \end{array},$$

with μ the location parameter (i.e., the chosen high threshold), σ the scale parameter, and ξ the shape parameter, which is related to the power exponent of Equation (16) via ξ=1/(α−1)=1/b. If ξ<0, the distribution has the upper bound $S_{max}=\mu +\sigma /\left|\xi \right|$. For the case ξ=0, the exponential distribution is retrieved [93]. It follows that

(24) $$\mathrm{Pr}\left(X>S\right)=\frac{1}{n}=\{\begin{array}{cc} \zeta {\left[1+\xi \left(\frac{S-\mu }{\sigma }\right)\right]}^{-\frac{1}{\xi }} & \mathrm{for}\;\xi \neq 0\\ \zeta \exp\left[-\frac{S-\mu }{\sigma }\right] & \mathrm{for}\;\xi =0 \end{array},$$

where “size” S is exceeded on average once every n observations (i.e., a return period) and where ζ=Pr(X>μ) is naturally estimated as the sample proportion of observations (or “events”) exceeding μ. The return level plot of the GPD is [93]

(25) $$S=\{\begin{array}{cc} \mu +\frac{\sigma }{\xi }\left[{\left(n\zeta \right)}^{\xi }-1\right] & \mathrm{for}\;\xi \neq 0\\ \mu +\sigma \log n\zeta & \mathrm{for}\;\xi =0 \end{array},$$

(see epidemic case below).

Since extreme values are commonly retrieved from individual sensors, the parameter set θ=(μ,σ,ξ) is usually site-specific. Parameter values are only provided below when associated to regional or global datasets (e.g., epidemics) and/or when a fit is provided in Figure 5:

- **Epidemics:** Considering a global dataset, [43] showed that epidemic sizes (per mil/year) follow a GPD (Equation (25)), with σ=0.0113 ‰/yr and ξ=1.40 for a fixed $\mu =10^{-3}$ ‰/yr in addition to ζ=0.38. We retrieved σ=0.0110 ‰/yr and ξ=1.41 (i.e., α=1.71 or b=0.71) for the same data and threshold (Figure 5).
- **River floods:** With the event size defined from the maximum discharge $\max Q_{p}$ observed in a year of daily measurements, flood sizes are described by the GEV distribution [111,112]. Taking the Potomac River dataset [46] as a textbook example, we obtained μ=2461.7 m^3^/s, σ=1171.7 m^3^/s, and ξ=0.19 (Figure 5). A power-law behavior has also been proposed [95].
- **Storms (tropical cyclones and other windstorms):** Both GEV and GPD distributions have been used to describe the size distribution of storms ($S=v_{max}$) and related perils. Parameterizations for specific cities and coastline segments can be found in the literature [113,114,115,116]. It can be noted that defining storm size in terms of total dissipation of power yields a power-law distribution with a relatively high exponent 2.28≤b≤4.15 [92]. Using such a proxy by summing over the cube of $v_{max}$ records per Δt interval for each track duration $\left[t_{0},t_{max}\right]$, i.e., $S=\overset{t_{max}}{\underset{i=t_{0}}{\sum }}v_{max,i}^{3}\Delta t$ [m^3^/s^2^] [79,92], we obtain b=3.01 for global data [48] (Figure 5).

### 2.4. Hazard Intensity Footprint

Once the size distribution $\lambda =f_{\lambda }\left(S\right)$ is defined for a given peril, the impact of each event on the environment must be assessed. We use the term *intensity* I to describe the physical quantity that leads to damage (i.e., a variable of the vulnerability function $f_{D}$). The area impacted by said intensity is known as the event *footprint*. A hazard intensity footprint I(x,y) can be modelled by following three main approaches: (i) an analytical expression of *spatial diffusion* with the value of I decreasing away from the event source (Section 2.4.1); (ii) a *threshold model* with I as a passive function of the variability on the underlying environment layer that is sliced (Section 2.4.2); or (iii) a numerical model of *dynamic propagation* of the event with I potentially increasing in the diffuse source (Section 2.4.3).

#### 2.4.1. Analytical Expressions of Static Event Spatial Diffusion

Although every hazard process is dynamical in nature, the process can be simplified to a static footprint where the event source is localized in most cases. For both point- and line-sources, the footprint is

(26) $$I\left(x,y\right)=f_{I}\left(S,r\right),$$

with (x,y) the spatial coordinates, S the event size, and r the distance to the event source. For track-sources, we can define

(27) $$I\left(x,y\right)=\underset{t}{\max}f_{I}\left(S,r,t\right),$$

which takes the maximum intensity observed at a given location over all timelapses t. For a point source, $r=\sqrt{{\left(x-x_{0}\right)}^{2}+{\left(y-y_{0}\right)}^{2}}$ with $\left(x_{0},y_{0}\right)$ as the source coordinates. For both line- and track-sources, $r=\underset{i}{\min}\sqrt{{\left(x-x_{0,i}\right)}^{2}+{\left(y-y_{0,i}\right)}^{2}}$ with $\left(x_{0,i},y_{0,i}\right)$ as the source coordinates (for an area source, these could correspond to the surface’s contour). Modifications may be applied to describe second-order variations in the footprint. Site-specific conditions can be modeled by simply adding a modifying factor to $f_{I}\left(S,r\right)$, as a function of (x,y). All the equations listed below are empirical relationships which are parameterized for some generic conditions. Epistemic uncertainties are not assessed but can be assumed to be large as only the simplest models are considered in this review. The reader is referred to the articles mentioned below for other functions, parameterizations, and calibrations.

**Figure 6:**
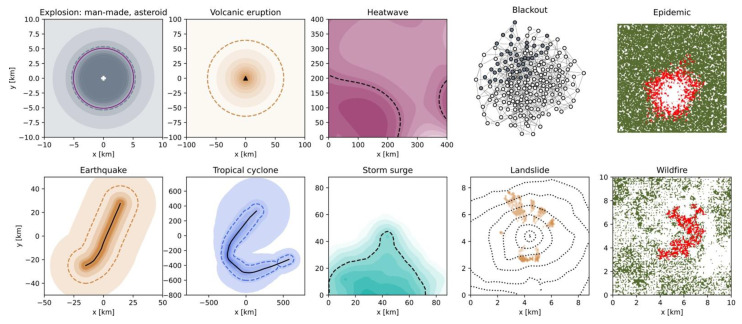
Examples of hazard intensity footprints based on analytical expressions (first two columns, Section 2.4.1), threshold models (central column, Section 2.4.2), and numerical models (last two columns, Section 2.4.3). DIFFUSION (POINT SOURCES)—**Explosion (incl. asteroid impact):** overpressure field p(x,y) for an ad-hoc $m_{TNT}=1$ Mton event (Equation (30)) with a 4-psi dashed contour in grey and matching 4-psi contour from Equation (28) in purple. **Volcanic eruption:** Ash depth map h(x,y) for a V=2.9 km^3^ (e.g., 1980, Mt St. Helens) and $h_{0}=1$ m event (Equation (33)) with a 5-cm dashed contour in orange. DIFFUSION (LINE SOURCES)—**Earthquake:** Peak ground acceleration footprint I(x,y) of an M=6.6 event on the ESHM13 fault segment ITCS073 with depth z=1 km (Equation 29) and a dashed 0.1-g contour in dark orange. DIFFUSION (TRACK SOURCES)—**Tropical cyclone:** Windspeed map v(x,y) for the IBTrACS 2005 Hurricane Katrina track (Equation (32) with $p_{n}=1005$ mb and $\log R=4.0441-1.2090\times 10^{-2}\left(p_{n}-p_{c}\right)+7.2694\times 10^{-3}\phi$ [161]) with a dashed 35-m/s wind speed contour in dark blue. THRESHOLD (AREA SOURCE)—**Heatwave:** Temperature map of Southwestern France at 3 pm on 17 July 2022 [44] with a dashed 35°-contour in dark magenta as (ad-hoc) proxy to the heatwave. THRESHOLD (LINE SOURCE)—**Storm surge:** Inundation map on a random topography (with fractal dimension $D_{f}=2.3$ [162]) with dashed 0-m altitude level as a coastline marker. DYNAMIC PROPAGATION (DIFFUSE SOURCES)—**Blackout:** Ring network with each node (power node or transmission line) connected to four others and with grey nodes as failed (i.e., blackout) following the rules of Equations (39) and (40). **Epidemic:** Infected and susceptible individuals colored in red and green, respectively, following the rules of Equation (42). **Landslide:** Event footprint formed of several patches, following the rules of Equations (43) and (44), on a random fractal topography (dotted contours with fractal dimension $D_{f}=2.3$ [162]). **Wildfire:** Burned areas and vegetation represented in red and green, respectively, following the rules of the Forest Fire model.

- **Asteroid (and comet) impacts:** The kinetic energy E of the celestial body transforms into destructive explosive energy at impact, which is described by peak overpressure p [psi]. The simplest approach consists of defining a binary intensity footprint, for instance
  (28) $$R=2.09h-0.449h^{2}E^{-\frac{1}{3}}+5.08E^{\frac{1}{3}},$$
  with R the 4-psi overpressure radius [km], E [Mton] the event impact energy, and h [km] the burst altitude [34]. Due to lack of data for calibration, the blast footprint formula is usually calibrated to data from nuclear tests [33,52,118] (see also the comparison between Equation (28) and the industrial explosion footprint case below and in Figure 6). Damage can also occur due to thermal radiation [34].
- **Earthquakes:** The general formulation of a ground motion prediction equation (GMPE) is
  (29) $$\log_{10}I=c_{1}+c_{2}M+c_{3}r+c_{4}\log_{10}r,$$
  with M the earthquake magnitude and r [km] the distance to the source [119,120]. The intensity I is often taken as the peak ground acceleration but can also be peak ground velocity, peak ground displacement, or spectral acceleration of felt intensity [121]. One of the simplest parameterizations is $c_{1}=-1.34$, $c_{2}=0.23$, $c_{3}=0$, and $c_{4}=-1$, with I as the PGA [g] and $r=\sqrt{d^{2}+z^{2}}$ [km], d being the distance to the surface projection of the fault rupture, and z the fault depth [122] (Figure 6).
- **Explosions (accidental or malicious):** A simple empirical relationship linking blast overpressure p [kPa] to the explosive mass $m_{TNT}$ [kg] is
  (30) $$p=\frac{1772}{r_{*}^{3}}-\frac{114}{r_{*}^{2}}+\frac{108}{r_{*}},$$
  with $r_{*}$ as the dimensional scaled distance according to Hopkinson–Cranz law $r_{*}=r/\sqrt[{3}]{m_{TNT}}$ and with r [m] as the distance to the source [123]. This is, for example, consistent with the binary footprint model (Equation (28)) proposed for asteroid impact CAT modeling (for an impact on the ground with h=0 and 1 psi = 6.89476 kPa). This is illustrated in Figure 6. The overpressure field for very large yields is calibrated to nuclear tests [118] and therefore also applicable to the blast component of nuclear explosions.
- **Tornados:** The mean wind field for a stationary tornado is calculated as the sum of the tangential and radial velocities $v_{tan}$ and $v_{r}$, with both wind velocity components v based on the Rankine vortex model,
  (31) $$v=\{\begin{array}{cc} v_{max}rR_{max}^{-1} & \mathrm{if}\;r\leq R_{max}\\ v_{max}r^{-1}R_{max} & \mathrm{if}\;r>R_{max} \end{array},$$
  with r the distance from the tornado origin, $v_{max}$ the maximum (tangential or radial) velocity, and $R_{max}$ the radius of the maximum (tangential or radial) velocity. An example of parameterization is $v_{tan,max}=60$ m/s, $v_{r,max}=30$ m/s, and $R_{max}=75$ m [124]. The intensity footprint of the tornado is obtained by adding the forward motion velocity $v_{f}$ to the wind field (e.g., $v_{f}=15$ m/s).
- **Tropical cyclones:** The wind profile of a tropical cyclone can be described by
  (32) $$v={\left(\frac{B}{\rho }\left(p_{n}-p_{c}\right){\left(\frac{R}{r}\right)}^{B}\exp\left[-{\left(\frac{R}{r}\right)}^{B}\right]+\frac{1}{4}r^{2}c^{2}\right)}^{\frac{1}{2}}-\frac{1}{2}rc,$$
  where r [km] is the distance to the cyclone center, R [km] the radius of maximum winds, and c [rad/s] the Coriolis parameter function of the latitude ϕ (see Equation (12) for the definition of $p_{n}$, $p_{c}$, ρ, and B) [78] (Figure 6). Nowadays, more sophisticated models are preferred [125], although Equation (32) remains important in CAT modeling conditional on the proper estimation of B [126].
- **Volcanic eruptions:** Apart from pyroclastic and lava flows, the principal hazard arises from the fall of airborne debris, ranging from blocks to ash, collectively known as tephra. The ash load is calculated by the pressure p=ρgh [Pa], where ρ= 900 kg/m^3^ is the density of dry ash and h is the ash layer thickness [m]. The ash thickness can be estimated from the exponential thinning law
  (33) $$h=h_{0}\exp\left(-\log(2)\frac{r}{R}\right),$$
  where $h_{0}$ [m] is the maximum thickness and R [m] is the half-distance (Figure 6). For a circular footprint,
  (34) $$R=\log\left(2\right)\sqrt{\frac{V}{2\pi h_{0}}},$$
  where V is the volume of tephra [127]. We remain unaware of any simple model to estimate $h_{0}$.

#### 2.4.2. Threshold Models of Passive Event Emergence

Some hazard intensity footprints can be generated by applying a threshold on an environment layer, such as a water level relative to sea surface on topography z(x,y) (storm surge case) or a temperature limit on the regional wet-bulb temperature $T_{W}\left(x,y\right)$ (heatwave case). A threshold model may have binary outcomes in which a value above threshold means potential harm and a value below none. The threshold defines the spatial contour of the hazard footprint.

- **Business interruptions:** There exists a lower damage threshold of ~5–10% that must be breeched to result in a business interruption, and an upper threshold, often as low as 50%, to cause the facility to completely shut down for repair or demolition [30]. This depends on the hazard intensity footprint of the trigger event.
- **Hail:** The contour of a convective storm is estimated from meteorological indicators. A hailfall footprint (often elliptical—see Section 2.2.3) then exists if the hailstone size (often assumed uniform in space) can exceed the threshold above which damage can occur (usually 2 cm in diameter) [128]. The hazard intensity I is then defined as the kinetic energy [J/m^2^]
  (35) $$E=c_{1}l^{c_{2}},$$
  with l the maximum hailstone diameter [mm] and empirical parameters of $c_{1}=0.3241$ and $c_{2}=1.843$ in [128].
- **Heatwaves:** Heat stress can be quantified by the wet-bulb temperature $T_{w}$, measured by covering a standard thermometer bulb with a wetter cloth and fully ventilating it. If $T_{w}$ exceeds a 35 °C threshold, hyperthermia follows [129]. The heat stress footprint can be derived from the temperature T map (which could be considered an unbounded area source; Figure 6) with the empirical expression
  (36) Tw=T·atan(0.151977ηw+8.313659)+atan(T+ηw)−atan(ηw−1.676331)+0.00391838ηw32·atan(0.023101ηw)−4.686035,
  where $\eta_{w}$ is the relative humidity [130].
- **Storm surges:** The so-called “bathtub” model defines a flooded area as all the locations below a certain elevation that are hydrologically connected to the coast, with the threshold based on the size of the storm surge event. In other words, it is a projection of a horizontal flood surface onto the topography (Figure 6). This model tends to overestimate flood extents [131]. More realistic models are based on hydrodynamics, a simplification of which are cellular automata (see Section 2.4.3). In this case, the discharge Q [m^3^/s] must be used as input, defined from
  (37) $$Q=hwv=hw\sqrt{gh},$$
  where h [m] is the height of water above the ground, w [m] the breach width, and v [m/s] the velocity of the water following the weir equation [29]. The breaching of a natural or man-made defense must also be modelled, which involves defense vulnerability analysis between event size assessment and flood modeling [29].

#### 2.4.3. Numerical Models of Dynamic Event Propagation

Some hazard processes cannot easily be simplified. This is especially true for perils with a diffuse source (see Section 2.2.5). Numerical modeling is then required to describe the dynamical process through time. In this case, energy does not dissipate but propagates through the extended source (and it is assumed that the event is extinguished immediately at the border of the source). A static footprint can still be defined with $I\left(x,y\right)=\underset{t}{\max}I\left(x,y,t\right)$. For the sake of simplicity and transparency, we here only consider cellular automata (CA) and their extension to agent-based models (ABM). Only their basic principles are explained. Other numerical methods could be used but cannot be condensed in this review. Most CA represent variants of the Sandpile model [132].

It should be noted that the intensity footprint of an event occurring on a diffuse source usually matches the event size S. The intensity is often binary: burned/not-burned in a wildfire, electricity off/on in a blackout, or people infected/not-infected in an epidemic, in which cases the intensity $\underset{x}{\sum }\underset{y}{\sum }I\left(x,y\right)=S$ is a count or an area. When the assets at risk are also equivalent to the source, there is no convolution operation needed between hazard and exposure. Modeling the hazard footprint is not required in this case, a good example being compartmental modeling for epidemics [133] where exposure, hazard, and losses are people. However, the hazard footprint remains a critical element for some other perils, for example landslides and river floods for which the intensity is defined as the soil thickness and water level h at (x,y), respectively. For those specific cases, numeric modeling is required to describe how mass movement occurs on an irregular surface (in disregard of the surface representing a diffuse source or not).

Numerical modeling of hazard footprints is necessary or often recommended for the following perils:

- **Armed conflicts (ABM):** A war is a cumulation of attacks and counterattacks, whose dynamics can be explained with Game Theory [36]. Although highly complex and heterogeneous in nature, some basic rules can be mentioned. The simplest model of attrition warfare is a set of ordinary differential equations (ODEs) defined as
  (38) $$\{\begin{array}{c} \frac{dN_{A}}{dt}=-c_{B}N_{B}\\ \frac{dN_{B}}{dt}=-c_{A}N_{A} \end{array}.$$
  where $N_{A}$ is the number of soldiers in the A army, each with offensive firepower $c_{A}$ (i.e., number of enemy soldiers killed per soldier from A), and $N_{B}$ is the number of enemy soldiers in the B army, each with offensive fire power $c_{B}$ (called Lanchester equations after F.W. Lanchester’s 1916 work) [134]. Solving Equation (38) indicates that the effectiveness of an army rises proportionally to the square of the number of its soldiers, but only linearly with their fighting ability. While Equation (38) is relevant for static trench warfare (see other ODEs in [135]), agent-based models can include spatial variations of forces, decision making, and psychology [136]. Agents are then combat units with a mission and situational awareness, among other characteristics. Their possible states S are alive, injured, or killed. The battlefield is the geographical lattice. However, the rules would be too numerous to list here [136,137]. A much simpler ABM for social unrest will later be provided that illustrates how different groups of individuals may act against each other [35]. The hazard footprint of an armed conflict would, in theory, be the sum of a heterogenous set of sub-footprints (e.g., explosion footprints—Equation (26), agents’ individual acts of violence—see social unrest and terrorist attack cases below, fires, etc.).
- **Blackouts (CA):** Cascading power failures can be modeled as a Sandpile on a network, instead of on a regular lattice. In the simplest generic configuration [138], each power line and generator have a region of safe operation, characterized by a load Z in a node. Links between nodes define the neighbors to which or from which a load increment is randomly transferred with
  (39) $$\{\begin{array}{c} Z_{i}\to Z_{i}\pm 1\\ Z_{j}\to Z_{j}\mp 1 \end{array}.$$
  when the power load exceeds the margin $Z_{c}$ at node i, then $N_{Z}$ units of load are transferred (and distributed randomly) to the failed node’s neighbors j:
  (40) $$\{\begin{array}{c} Z_{i}\to Z_{i}-N_{Z}\\ Z_{j}\to Z_{j}+n_{Z,j} \end{array},$$
  with the condition $\underset{j}{\sum }n_{Z,j}=N_{Z}$. The Sandpile network self-organizes, with cascading failures potentially leading to large-scale blackouts [138]. The footprint of the blackout is then defined as all the nodes that failed in one event (Figure 6).
- **Crop failures (due to pests, ABM):** Pest dynamics can be described by a set of ODEs that describes inter-species interactions. They can be multiple and play at different spatiotemporal scales in an ecosystem. The simplest model is the predator–prey Lotka–Volterra model [139,140]
  (41) $$\{\begin{array}{c} \frac{dN_{prey}}{dt}=c_{1}N_{prey}-c_{2}N_{prey}N_{pred}\\ \frac{dN_{pred}}{dt}=c_{3}N_{prey}N_{pred}-c_{4}N_{pred} \end{array}$$
  where $N_{prey}$ is the prey density (e.g., the crops) and $N_{pred}$ the predator density (e.g., the pest). Note the resemblance to the model of attrition warfare (Equation (38)). A far more complex model is the seminal ‘insect outbreak system’ of [141], which—in its simplest form—describes interactions between a pest (the spruce budworm), its predator (some birds), and the exposed vegetation (the forest). Several CA and ABM have been developed for insect pest assessment [32]. Simple agent rules can be derived from Equation (41) by, for instance, adding a spatial component, random agent movement, and a contact radius. The intensity of an event could be defined by the aggregate size of the pest on the crops, $S=N_{pred,max}$, or the direct damage, $N_{prey,min}$.
- **Cyber-attacks (various):** Cyber-catastrophes propagate via cascading effects within IT systems and networks. For data exfiltration cases, the final event size, or event footprint extent, can be defined on a data breach severity scale function of the number of lost personal records N (P3, for the range 1000–10,000, to P9, for N> 1 billion [22]). Cyber-attacks may also cascade into critical infrastructure failures (e.g., blackout—see above) and socio-economic events [22]. Their footprints (both virtual and physical) are highly scenario-dependent. However, [22] indicated a 1.6 economic multiplier when considering loss increase due to cascades in a trading network of companies. The dynamics of a cyber-attack is mainly governed by the principle of least action, i.e., striking targets with inferior security, and follows the rules of Game Theory [22]. Various statistical models have been proposed [21,142] which are outside the scope of this paper. On the physical side, epidemic models, for example (see below), have been modified to quantify the spread of a piece of malware [143].
- **Epidemics (ABM):** The simplest epidemic model is the Susceptible-Infectious-Recovered (SIR) model [133]. Many more sophisticated models exist [144,145] that derive from the SIR set of ODEs:
  (42) $$\{\begin{array}{c} \frac{dS_{S}}{dt}=-\beta S_{S}S_{I}\\ \frac{dS_{I}}{dt}=\beta S_{S}S_{I}-\frac{1}{\Delta t}S_{I}\\ \frac{dS_{R}}{dt}=\frac{1}{\Delta t}S_{I} \end{array},$$
  where $S_{S}$ is the susceptible stock, $S_{I}$ the infected stock, $S_{R}$ the recovered stock, and $N_{tot}=S_{S}+S_{I}+S_{R}$ (see Section 2.2.5 and Equation (13) for the definition of β and Δt). The controlling parameter is the basic reproduction number $R_{0}$ (Equation (13)); a feedback loop leading to an epidemic occurs for $R_{0}>1$. While Equation (42) can be solved by numerical integration, agent-based models attempt to capture the real-world heterogeneous mixing of agents [146,147]. Each stock, or compartment, then represents a state. In the simplest configuration, agents move randomly and, if an infected agent is within infection range of a susceptible agent, $S_{S}\to S_{I}$. $S_{I}\to S_{R}$ after Δt (Figure 6). Such a model can incorporate local knowledge of demographic data, the healthcare system, and human contact networks [24].
- **Floods (river flood, storm surge, tsunami—CA):** Flood intensity usually refers to the inundation depth h. Although h depends on the peak discharge $Q_{p}$ and the shape of the valley [148], modeling is required to properly consider the variations in topography. A simple CA can be defined with the following rules:
  1. Define the absolute height (or motion cost) as the sum of the altitude and water height $h_{tot}=z+h$;
  2. Calculate the gradient (or weight) between the central cell and von Neumann neighbor cells (zero weight for neighbors with equal or greater $h_{tot}$);
  3. Discharge the central cell with (some of) the water distributed to the neighbor cells, depending on their weight.
  The first discharge occurs at the source of the flood, with $h=Q_{p}\Delta t/w^{2}$ where Δt is the time interval between two steps and w the cell width. The motion cost can include soil characteristics, such as roughness and infiltration potential. The weights for water distribution are a function of the motion cost at the central and neighbor cells [149], which, in the simplest case, is proportional to the normalized gradients. A similar CA strategy can apply to tsunamis [150].
- **Landslides (CA):** The propagation of a landslide can be modelled as a Sandpile with the environment—or diffuse source—defined by the topography z(x,y) and the soil thickness h(x,y). The simplest case consists of initiating mass movement in cells $\left(x_{0},y_{0}\right)$ of unstable slope, which is defined by $F_{S}\left(x,y\right)<1$ (i.e., seed event, see Equation (14)) [151]. The mass is transferred downward to the Moore neighbor of maximum gradient $\left(x_{1},y_{1}\right)$, so that
  (43) $$\{\begin{array}{c} \begin{array}{c} z\left(x_{0},y_{0}\right)\to z\left(x_{0},y_{0}\right)-\Delta h\\ h\left(x_{0},y_{0}\right)\to h\left(x_{0},y_{0}\right)-\Delta h \end{array}\\ \begin{array}{c} z\left(x_{1},y_{1}\right)\to z\left(x_{1},y_{1}\right)+\Delta h\\ h\left(x_{1},y_{1}\right)\to h\left(x_{1},y_{1}\right)+\Delta h \end{array} \end{array},$$
  and with mass movement defined, for example, by
  (44) $$\Delta h=\min\left(\frac{1}{2}\left[z\left(x_{0},y_{0}\right)-z\left(x_{1},y_{1}\right)\right]-w\tan\phi_{stable},h\right)$$
  with $\phi_{stable}$ the maximum slope angle ϕ for which $F_{S}\geq 1.5$. Since $h\left(x_{1},y_{1}\right)$ increases, $F_{S}\left(x_{1},y_{1}\right)$ can cross the instability threshold, hence further propagating the landslide (Figure 6). Many model variants exist [152,153,154,155]. Note that the landslide source was previously defined as diffuse instead of an area source because part of the soil outside the seed event may participate in landslide propagation (Equation (43)).
- **Social unrest (ABM):** A simple model of civil violence [35] consists of two types of agents: population and cops. Population agents can be in one of three states (quiet $S_{Q}$, active $S_{A}$, or jailed $S_{J}$). They have a fixed degree of grievance $C_{G}$, a fixed degree of risk aversion $C_{RA}$, and a vision radius $r_{v,pop}$. Cops have a vision radius $r_{v,cop}$. All agents are also characterized by their location (x,y). There are three rules:
  1. *General rule:* Move to an empty cell (or where someone is jailed);
  2. *Population rule:* If $C_{G}-C_{NR}>T$, become active ($S_{Q}\to S_{A}$), otherwise stay quiet ($S_{Q}$);
  3. *Cop rule:* arrest a random active agent located within $r_{v,cop}$ ($S_{A}\to S_{J}$)
  where $C_{NR}=C_{RA}P$ is the net risk and T a threshold for rebellion.
  (45) $$P=1-\exp[-c\frac{N_{cop}}{N_{A}+1}]$$
  is the arrest probability, which depends on the number of cops $N_{cop}$ and the number of active agents $N_{A}$ observed within $r_{v,pop}$ (and with c as a normalization constant). For each jailed agent, the jail term is random and uniform in the range $\left[0,\Delta t_{J,max}\right]$, with $S_{J}\to S_{Q}$ once released. Due to the form of Equation (45), a contagion process can occur, leading to a large-scale riot [35] whose intensity could, in theory, be defined by the total number of violent individuals $N_{A,max}=S$. The duration of the riot could be a second measure of hazard intensity.
- **Terrorist attacks (ABM):** Large-scale terrorist attacks generally infer the use of explosives (see above). The choice of location for an attack can be explained by Game Theory, but the modeling of agents is not required. In other types of attacks, such as a group of terrorists attacking civilians with knifes, an ABM can be formulated. Ref. [156], for example, combined the effect of such an attack with the risk of stampede in a closed environment. Terrorists search targets in their radius of vision, while civilians attempt to flee with direction and speed depending on the amount of blood lost and collisions with other agents. Variants are too numerous to mention any specific model in the context of this review.
- **Wildfires (incl. urban fires, CA):** The so-called Forest Fire model is defined by four rules:
  1. An empty space fills with a tree with probability $P_{tree}$ (i.e., tree growth);
  2. A tree ignites due to a lightning strike of probability $P_{fire}$;
  3. A tree burns if at least one von Neumann neighbor is burning;
  4. A burning cell turns into an empty cell.

A binary footprint is defined from the clusters of burned cells (Figure 6). Standard models will include wind direction and windspeed, relative humidity, fuel moisture content, air temperature, and topography [157,158,159]. More sophisticated physics-based models will also include radiation, convection, conduction, and other processes, which can, for example, describe the pyrocumulus phenomenon associated to some mega-fires [160]. The general rules previously described can also apply to urban fires under different parameterizations [28].

## 3. Peril Harmonization via the Concept of Energy Transfer

**Figure 7:**
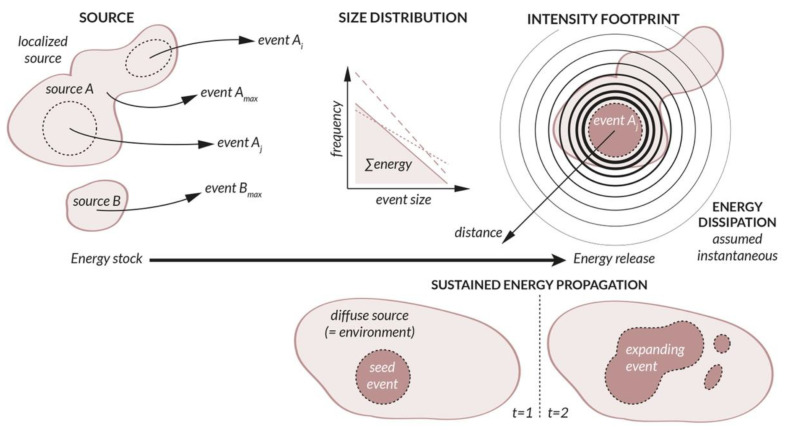
Sketch describing the hazard process in terms of energy transfer, applicable to any peril. See text for details.

The present review illustrated the heterogeneity of the physical processes involved in the occurrence of different perils (e.g., Figure 6). Although they can be grouped by source type (Section 2.2), size distribution type (Section 2.3), and intensity footprint type (Section 2.4), event size metrics are not intelligibly exchangeable. An obvious choice for a common size measure is the energy being released by an event [163,164]. We can indeed describe the hazard process in terms of energy transfer, as illustrated in Figure 7 (note that power could be another option). Following the hazard pipeline of standard CAT modeling, the source represents the energy stock, the size distribution indicates how energy is released via the emergence of events, and the intensity footprint characterizes how energy dissipates in the environment. For a localized source, the energy released can only diffuse in space from the region of high energy concentration that is the source to the surrounding environment. For a diffuse source, the energy released by the initial event (or seed event) can transfer to the environment, which acts as a continuous energy stock promoting sustained energy propagation. In this case, event size and intensity footprint are virtually indistinguishable. Nonlinear dissipative systems are known to release energy sporadically by following a power-law size distribution (Figure 5). This is a property of systems in the state of self-organized criticality, which is exemplified in the Sandpile CA [132] and believed to occur for many perils [154,165,166].

Johnston [164] devised a ranking of selected historical events using energy as a common metric, comparing past earthquakes, volcanic eruptions, tropical cyclones, tornadoes, landslides, and nuclear explosions, as well as a lightning bolt on the low-energy side and a hypothetical 10-km asteroid impact on the high-energy side. Despite the importance of such a comparison, this work has only been cited 10 times over the past 30 years (August 2022 Google Scholar search), suggesting that peril harmonization via energy has yet to be addressed.

Energy can take many forms, which can all be categorized as kinetic energy (motion) or potential energy (stored). Kinetic energy includes radiant (electromagnetic) energy, thermal (heat) energy, motion energy, wave energy, and electrical energy. Potential energy includes chemical energy, mechanical energy, nuclear energy, and gravitational energy. For natural perils, and some man-made ones, damage is due to destructive energy. However, for some man-made perils, the damage can be a lack of energy that is needed for the functioning of society [167], since energy is fundamentally the ability to work. The energy transforms from when it is stored in an event source to when it is released in the environment, with part of the original energy always converted to heat. Table 1 lists the perils considered in this study and the main energy form(s) they take. This is indicative in nature, as a review of the proportion of energy types per peril is outside the scope of the present invited article. For example, for asteroid impacts, a thermal radiation model can be coupled to the blast model [34], consistent with models of nuclear explosions [118]. For floods, both water height h (potential energy) and flow velocity v (kinetic energy) lead to damage, which can be combined with the formula $h+v^{2}/\left(2g\right)$ [168]. For volcanic eruptions, thermal energy depends on mass erupted, which is proportional to the volume V [64], etc.

**Table 1:** Energy types per peril (indicative only, non-exhaustive).

Peril	Event Size S (Section 2.2) → Intensity I (Section 2.4)	Matching Energy Types
Armed conflicts	Various, so far aggregated in terms of loss L (Equation (38))	Various, aggregation TBD ^1^
Asteroid impacts	Kinetic energy E (Equation (2)) → overpressure p (Equation (28))	Motion → wave (air) (+radiant, thermal)
Blackouts	E.g., unsupplied electrical energy E (Equation (40))	Electrical (*lack of*)
Business interruption	Revenue loss L	Work done (*lack of*)
Crop failures	So far in terms of farming production yield loss L (Equation (41))	Chemical (food) (*lack of*)
Cyber-attacks	E.g., number of data breaches N	Stored information (*lack of*)
Earthquakes	Magnitude M (Equations (7) and (8)) → PGA (Equation (29))	Mechanical (elastic) → wave (seismic)
Epidemics	Infection count N (Equation (42))	TBD ^1^
Explosions (nuclear)	Explosive yield E (Equation (4)) → overpressure p (Equation (30))	Nuclear → wave (air) (+radiant, thermal)
Explosions (other)	TNT mass m (Equation (3)) → overpressure p (Equation (30))	Chemical → wave (air) (+thermal)
Floods	Discharge Q (Equations (5) and (37)) → water depth h	Motion + gravitational → gravitational (+motion)
Hail	Hailstone diameter l → kinetic energy E (Equation (35))	Gravitational → motion
Heatwaves	Temperature T	Thermal
Landslides	Area A or volume V → soil height h	Gravitational → gravitational (+motion)
Social unrest	Number of violent individuals N as possible proxy	Various (thermal via arson, mechanical)
Storms	Windspeed v (Equation (12)) → Equation (32))	Motion (+water latent heat) → motion
Tsunamis	Potential energy E (Equation (10)) → Water height h	Gravitational → wave (water) (+motion)
Volcanic eruptions	Erupted volume V → ash depth h (Equation (33))	Thermal → gravitational (+thermal)
Wildfires (incl. urban)	Burnt area A	Thermal (+radiant)

^1^ Debatable when size and/or intensity are defined in terms of human losses—see text below for a discussion.

Human losses may be used to describe the size and/or intensity of an event, for example the number of people infected in an epidemic (which is proportional to fatalities in the risk component of a CAT model). Heterogeneous types of destructive energy in armed conflicts, terrorism, and social unrest are also aggregated in terms of human losses in practice. Relating human loss to energy is, however, tricky. The metabolic heat of a resting human body could represent the lower bound of the energy loss (~100 W of power × lifespan [129]). If individuals are considered as workers in a society, their loss could be defined in terms of lack of work related to muscle power or to their amplified power by machines [169]. Their role may not be actual physical work but intellectual work, with a connection to be made between information (as entropy) and energy [169,170]. Note that an information-based metric could also be used to describe energy loss in a cyber-attack (Table 1). A life has obviously far more value, which might also be related to information content, such as memories defining part of an individual’s identity and character. These considerations significantly stretch the idea of using energy as a simple metric to compare different perils, but they are worth pointing out.

We already defined event size or hazard intensity in terms of energy for several perils (see Equations (2)–(4), (10) and (35) and Table 1). A dimensional analysis shows how different physical parameters of a hazard process can be combined to define S or I in terms of energy, which has dimension [M^1^L^2^T^−2^]. Ref. [79] did so for hurricanes, considering power instead of energy. Ref. [72] reviewed methods to assess the energy released by tsunamis and used dimensional analysis to calculate their power. For wildfires, the Stefan–Boltzmann constant shows that it must be multiplied by burning area A and the fourth power of temperature, $T^{4}$, to connect to a radiative energy measure [171]. The reader can do such an analysis for many of the perils physically described in this review. Although the standard International System of Units for energy is the Joule, it is convenient for catastrophes to instead use the explosive power equivalent to one ton of TNT (trinitrotoluene) (e.g., Equation (3)), which converts to about $4.184\cdot 10^{9}$ Joules.

## 4. Conclusions

This review is the first to consider so many perils while providing all the equations necessary for basic hazard assessment in the context of CAT risk modeling. By categorizing perils per event source type (Section 2.2), size distribution (Section 2.3), and intensity footprint modeling strategy (Section 2.4), we were able to describe a heterogeneous collection of physical and environmental processes within a common scheme. Different perils can now be considered different flavors of what we may refer to as the *catastrophe object* (Figure 7). This ontological analysis shall help with the development of CAT modeling as a distinct scientific discipline [4]. By describing any peril in terms of event size S, size distribution Pr(S), and intensity footprint $I\left(x,y\right)=f_{I}\left(S\right)$, peril-specific jargon and methods can be minimized, and silo effects hence reduced. It should also facilitate the implementation of emergent risks by directly following the CAT modeling paradigm.

One of the main goals of this study was to describe as many perils as possible, but some are still missing. Those include droughts, toxic release, and financial crises among others. They could, however, be implemented within the proposed classification strategy in the future. Although most of the presented models remain very simple, they can be used as a basis for the development of more sophisticated and more realistic models. These hazard pipeline templates should help the student of risk to create new CAT models from the fundamental concepts and laws of CAT risk science. This study has also the potential to foster multi-risk modeling [172,173]. Ready-made models for basic hazard assessment indeed provide the inputs necessary to implement more peril interactions than are usually performed. In regard to global warming, the availability of simple models for many climate-related perils should also facilitate the implementation of more of them in climate risk modeling frameworks [16,174] and related system dynamics models [175]. Since global warming means more energy being transferred into the atmosphere, our approach of peril harmonization via the concept of energy transfer remains fully valid. Overall, the results of this review will be helpful for the prototyping of more complex risk problems.

Finally, based on the synopsis provided in Section 2 and the discussion on energy transfer in Section 3, it is now possible to compare and rank many perils, à la Johnston [164]. This will be the topic of a future article.

## Data Availability

All the data used in this study are publicly available, or otherwise available upon request from their authors (as per the given references).

## Appendix A

**Table A1:** List of symbols used in the present article.

Symbol	Dimension	Description
a	various	Productivity parameter of the power-law
A	[L^2^]	Area
b	[1]	Power-law exponent in cumulative form (=α−1)
B	[1]	Holland parameter for tropical cyclones
c	[1]	Empirical parameter, scaling factor
C	[ML^−1^T^−2^]	Soil cohesion
d	[L]	Distance
D	[1]	Damage, e.g., mean damage ratio
e	[1]	Euler’s number (≈2.718)
E	[ML^2^T^−2^]	Energy
$f_{D}$	-	Damage function $f_{D}\left(I,\cdots \right)$
$f_{I}$	-	Intensity function $f_{I}\left(S,r,\cdots \right)$
$f_{\lambda }$	-	Frequency function $f_{\lambda }\left(S\right)$
$F_{S}$	[1]	Factor of safety for landslides
g	[LT^−2^]	Gravitational acceleration (≈9.81 m/s^2^)
h	[L]	Height, depth
I	various	Intensity of event
l	[L]	Length, diameter
L	various	Loss (e.g., economic, human)
m	[M]	Mass
M	[1]	Magnitude of earthquake
$M_{0}$	[ML^2^T^−2^]	Seismic moment
n,N	[1]	Number, count
p	[ML^−1^T^−2^]	Pressure, overpressure
pr, Pr	[1]	Probability (non-cumulative, cumulative)
Q	[L^3^T^−1^]	Water discharge
r,R	[L]	Radius, radial distance
$R_{0}$	[1]	Epidemic basic reproduction number
S	various	Size of event
t	[T]	Time increment
T	[Q]	Temperature
u	[L]	Displacement
v	[LT^−1^]	Velocity
V	[L^3^]	Volume
w	[L]	Width
x,y,z	[L]	Geographical coordinates
X	various	Random variable
Z	various	Electricity load
α	[1]	Power-law exponent (=b+1)
β	[T^−1^]	Infectious disease transmission parameter
Δh	[L]	Thickness
ΔH	[L^2^T^−2^]	Heat of combustion
ΔT	[T]	Return period, time interval
ε	[1]	Eccentricity
ζ	[1]	Pr(X>μ) in GPD
η	[1]	Fraction, ratio
θ	various	Parameter set
λ	[T^−1^]	Rate of occurrence
μ	various	GEV and GPD location parameter
ξ	[1]	GEV and GPD shape parameter
ρ	[ML^−3^]	Density
σ	various	GEV and GPD scale parameter
τ	[T]	Time
ϕ	[1]	Angle
$C_{i}$	various	Characteristics of an agent
$S_{i}$	-	State of an agent
T	various	Threshold

**Table A2:** Color scheme per peril category.

Color	Category	Perils
■	Climatological	Heatwave
■/■^1^	Ecological	Crop failure, epidemic, wildfire
■	Extraterrestrial	Asteroid and comet impact
■	Geophysical	Earthquake, landslide, volcanic eruption
■	Hydrological	River flood, storm surge, tsunami
■	Meteorological	Convective storm (incl. hail, tornado, lightning), (extra-)tropical cyclone, other storms
■	Socio-economic	Armed conflict, social unrest, terrorism
■	Technological	Blackout, cyber-attack, explosion, fire

^1.^ For footprints, ecological environment in dark olive green and active footprint in red (see Figure 6).
